# Pill or procedure? Patient preferences on beta-blockers as an alternative to endoscopic variceal screening during the COVID-19 pandemic

**DOI:** 10.1093/gastro/goac015

**Published:** 2022-05-06

**Authors:** Zheng Che, Jeremy Louissaint, Ihab Kassab, Elliot B Tapper

**Affiliations:** 1 Department of Internal Medicine, University of Michigan, Ann Arbor, MI, USA; 2 Division of Gastroenterology and Hepatology, University of Michigan, Ann Arbor, MI, USA; 3 Gastroenterology Section, VA Ann Arbor Healthcare System, Ann Arbor, MI, USA

## Introduction

Certain patients at low risk of clinically significant esophageal varices by Baveno criteria can safely avoid screening esophagogastroduodenoscopy (EGD) [1, 2]. However, regardless of variceal status, the PREDESCI study suggests that earlier beta-blocker (BB) use in compensated disease with clinically significant portal hypertension by hepatic venous pressure gradient may result in benefit by delaying the time to decompensated cirrhosis [3, 4]. This carries significant ramifications for future clinical guidelines and decision-making for whom we decide to initiate BB.

The COVID-19 pandemic has raised challenges for procedure units resulting in EGD delays and case backlogs, which has resulted in a consensus statement urging providers to consider earlier initiation of pharmacologic variceal prophylaxis instead of EGD in appropriate populations [5, 6]. While expanding BB prophylaxis in select patients who are unable to obtain a timely EGD is attractive, patient acceptance and perspectives regarding this are unknown. Pre-primary BB use without EGD can only be effective in the setting of consistent medication adherence, which necessitates a basic understanding of patient preference and perspectives.

## Methods

### Patient selection

We generated a survey cohort using a data repository at our tertiary center. Our inclusion criteria included adult patients (≥18 years) recently seen in our hepatology clinic for cirrhosis care with a platelet count of <150,000 and a valid email address for recruitment. Manual chart review of respondents was performed to confirm inclusion criteria and to collect demographic data.

### Patient survey

We utilized Research Electronic Data Capture (RedCap) for survey creation and email distribution. A total of 944 surveys were sent, with a response rate of 21.5% (203 patients). The survey began with a summary of our current screening paradigm and standard of care for esophageal varices, and of ongoing discussions that the pre-primary use of BB (in lieu of EGD first for refinement of use) may be appropriate in select patients. Survey questions utilized Likert-scale scoring to investigate patient preferences surrounding BB, EGD, and concerns related to COVID-19 (Supplementary Figures 1 and 2).

### Data analysis

Our primary outcome was patient preference to trial BB first over EGD screening, which was asked as “I would like to try beta blocker first and then decide.” Multinomial logistic regression was also performed to assess which factors were associated with willingness to trial BB first vs EGD first based on patient-reported preferences. Data are presented as odds ratios, utilizing neutral responses as the reference group. Covariates examined included sex, Model of End-stage Liver Disease-Sodium (MELD-Na) score, exposure histories (previous EGD, BB use, adverse effects to BB, variceal bleed), as well as general perceptions surrounding EGD, BB, variceal bleeding, and COVID-19.

## Results

Our surveyed cohort demographics (Supplementary Table 1) included a mean MELD-Na of 11. The results showed that 91.6% had prior EGD(s) for any reason and 53.7% had previous or current BB use for any reason. Overall, 29.1% had compensated disease.

**Figure 1.**
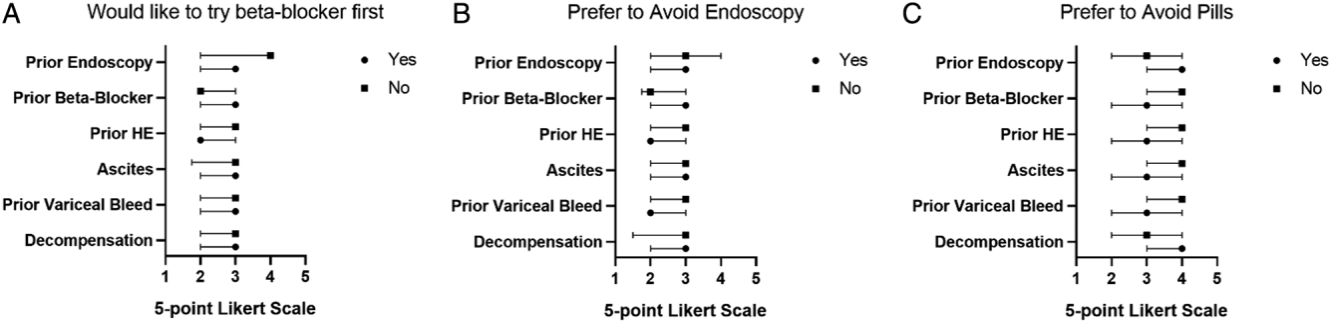
Comparison of Likert-scale responses among demographic subgroups. Likert-scale responses were recorded on the *x*-axis for each question, with 1 = strongly disagree, 2 = disagree, 3 = neutral, 4 = agree, and 5 = strongly agree. Questions were “I would like to try a beta blocker first” (A), “Avoiding endoscopy is important to me” (B), and “Avoiding pills is important to me” (C). Subgroups analysed are shown on the *y*-axis. Decompensation was defined as the presence of one or more of prior variceal bleed, hepatic encephalopathy, or ascites. HE, hepatic encephalopathy.

Survey results showed that 20.2% of patients preferred BB first, 45.8% were neutral, and 34.0% favored EGD first. While only 12.5% felt that avoiding EGD was important (30.5% neutral), 50.2% felt that avoiding another pill was important (25.6% neutral), 44.8% were concerned about BB side effects (28.1% neutral), and 37.9% stated that they preferred avoiding the hospital due to COVID-19 (21.5% neutral). In Figure 1, we contrast unadjusted preferences for choosing pre-primary BB over EGD and avoidance of EGD or pills across the clinical subgroups. While some differences exist, disease severity and prior treatment exposures did not significantly influence preferences, with significant neutrality expressed. Notably, preferences were not impacted by decompensated disease.

We analysed the impact of our covariates and Likert-scale questions surveyed on willingness to trial pre-primary BB by multinomial logistic regression (Table 1). A preference for BB first was significantly associated with a desire for personalized physician recommendations, even if differing from current guidelines [odds ratio (OR), 1.87; 95% confidence interval (CI), 1.16–3.01]. Those with a prior EGD were significantly less likely to prefer BB first (OR, 0.11; 95% CI, 0.02–0.52). Factors significantly associated with preference for EGD included a desire to avoid pills (OR, 1.41; 95% CI, 1.08–1.85) and concern for BB side effects (OR, 1.56; 95% CI, 1.17–2.09). Concern for hospital-related COVID-19 exposure (OR, 1.21; 95% CI, 0.89–1.65) did not exhibit statistical significance compared with neutral respondents.

**Table 1.** Multinomial logistic regression of surveyed factors and preference for endoscopy or beta blocker first

Factor	EGD first	BB first
Male	0.96 (0.52–1.80)	1.22 (0.56–2.67)
MELD-Na (per 1-point increase)	0.98 (0.91–1.05)	0.97 (0.88–1.06)
Had prior EGD (Yes)	0.43 (0.08–2.21)	0.11 (0.02–0.52)^b^
Had prior variceal bleed (Yes)	0.94 (0.47–1.85)	1.32 (0.58–2.98)
Had prior BB use (Yes)	0.47 (0.25–0.89)^b^	0.73 (0.33–1.60)
Self-assessed risk of variceal bleed (per 10% increase)	0.95 (0.84–1.08)	1.01 (0.86–1.18)
Want to avoid pills (per 1-point increase^a^)	1.41 (1.08–1.85)^b^	0.95 (0.69–1.30)
Want to avoid EGD (per 1-point increase^a^)	0.63 (0.47–0.85)^b^	1.40 (0.99–1.97)
Concerned about EGD risks (per 1-point increase^a^)	0.74 (0.55–0.99)	1.24 (0.89–1.75)
Concerned about BB risks (per 1-point increase^a^)	1.56 (1.17–2.09)^b^	1.05 (0.75–1.48)
Concerned about COVID risks (per 1-point increase^a^)	0.86 (0.67–1.10)	1.21 (0.89–1.65)
Want personalized recommendations (per 1-point increase^a^)	1.12 (0.82–1.54)	1.87 (1.16–3.01)^b^

EGD, endoscopy (esophagogastroduodenoscopy); BB, beta blocker; MELD-Na, Model of End-stage Liver Disease-Sodium.

All values are presented as odds ratio followed by 95% confidential interval in parentheses; reference: neutral response.

^a^ Likert scale from 1 to 5 with 1 = strongly disagree, 3 = neutral, and 5 = strongly agree.

^b^ Denotes statistical significance.

## Discussion

Pre-primary BB use for select patients at low risk of varices requiring treatment is attractive in the context of emerging data suggesting the ability to prevent not only variceal bleeding but also overall hepatic decompensation [3, 4]. The backlog of procedures due to COVID-19 forces prioritization decisions [5] and PREDESCI suggests that there are long-term benefits conferred from early BB initiation [3]. There is enthusiasm for the implementation of pre-primary BB in the scientific community, although its success will be dependent on patient factors.

Our survey indicates that the majority of our cohort is either neutral or agreeable to pre-primary BB. Factors decreasing BB acceptance include the preference of avoiding medications and concern for BB side effects. Notably, COVID-19 did not have a statistically significant impact on preferences, suggesting that shared decision-making may be less effective if centered on COVID concerns. Above all, the desire to receive personalized physician recommendations, even if this differs from current guidelines, persuades many patients. This emphasizes how critical counseling and shared decision-making are in the context of significant neutrality.

Our data identify key patient characteristics that influence preference for BB use but must be interpreted within the context of the study design. The response rate was limited by certain survey factors. We note that some participants did not ultimately complete the survey, which may have been due to the moderately lengthy educational component, as well as the number of questions. The lower response rate may affect the overall generalizability and reliability of statistical significance. Many patients also had prior exposure to EGD and it is thus unclear how these findings generalize to the rare persons without EGD experience. Future directions include survey recruitment of patients with a new or recent diagnosis of cirrhosis without prior exposures to BB or EGD to remove those influences, including a possible prospective study design.

## Conclusions

Most patients we surveyed are neutral to pre-primary BB for variceal bleed prophylaxis, which is attractive during the current pandemic and beyond. Our data indicate that patients who are highly concerned about pill burden and BB side effects may be less suitable for a BB-first strategy, though all patients would benefit from a direct explanation of the rationale by their trusted provider.

## Supplementary Data

Supplementary data is available at *Gastroenterology Report* online.

## Authors’ Contributions

Z.C. and E.B.T. conceptualized and designed the study. Z.C. performed study implementation. Z.C. and I.K. participated in data acquisition. Z.C., J.L., and E.B.T. performed data analysis and interpretation. Z.C. drafted the manuscript. All authors read and approved the final manuscript.

## Funding

E.B.T. is supported by NIDDK K23 DK117055.

## Conflict of Interest

E.B.T. has served on advisory boards for Mallinckrodt, Kaleido, Rebiotix, Novo Nordisk, and Bausch Health; consulted for Allergan, Novartis; and has received unrestricted research grants from Valeant, Gilead. All other authors have none declared.

## Supplementary Material

goac015_Supplementary_Data
