# Supplementary material for: Pill or procedure? Patient preferences on beta-blockers as an alternative to endoscopic variceal screening during the COVID-19 pandemic
Source: Gastroenterol Rep (Oxf). 2022 May 6;10:goac015. doi: 10.1093/gastro/goac015 (PMC9073924; doi:10.1093/gastro/goac015)
Supplement: goac015_Supplementary_Data [file goac015_supplementary_data.zip › 2022-042 Supplementary materials.docx]

**Supplementary Table 1.** Cohort demographics of 203 patients with cirrhosis

| Characteristic | Value |
| --- | --- |
| Mean age, years (STD) | 60 (12.1) |
| Female, *n* (%) | 93 (45.8%) |
|  |  |
| **Cirrhosis etiologies**, *n* (%) |  |
| Alcohol | 33 (16.3%) |
| Non-alcoholic fatty liver disease | 70 (34.5%) |
| Hepatitis C | 26 (12.8%) |
| Hepatitis B | 7 (3.4%) |
| Primary biliary cholangitis | 8 (3.9%) |
| Primary sclerosing cholangitis | 10 (4.9%) |
| Cryptogenic | 10 (4.9%) |
| Multiple etiologies, other | 36 (17.7%) |
|  |  |
| **Cirrhosis complication(s) and care**, *n* (%) |  |
| Compensated disease* | 59 (29.1%) |
| Ascites | 103 (50.7%) |
| Spontaneous bacterial peritonitis | 23 (11.3%) |
| Hepatic encephalopathy | 75 (36.9%) |
| Variceal bleed | 63 (31.0%) |
| Previous endoscopy | 186 (91.6%) |
| Previous beta blocker use | 109 (53.7%) |
|  |  |
| **Laboratory data, mean (STD)** |  |
| Platelet count, (10^3^/µL) | 101.9 (40.5) |
| Albumin level (g/dL) | 4.2 (3.9) |
| MELD-Na score | 10.7 (4.3) |
|  |  |

STD, standard deviation.

*****Absence of hepatic encephalopathy, ascites, or variceal bleed


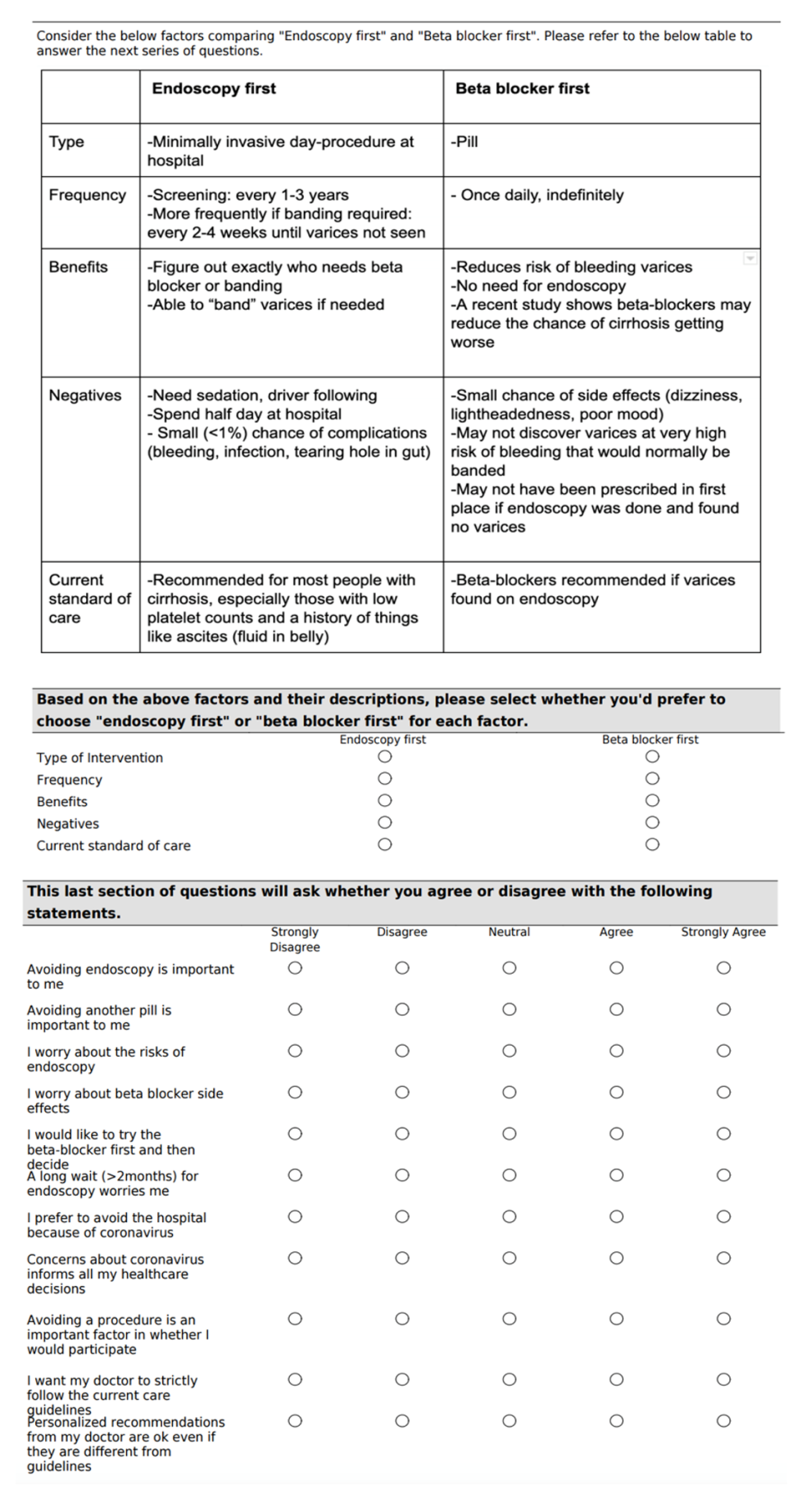


**Supplemental Figure 1**. Patient Survey – Table and Questions


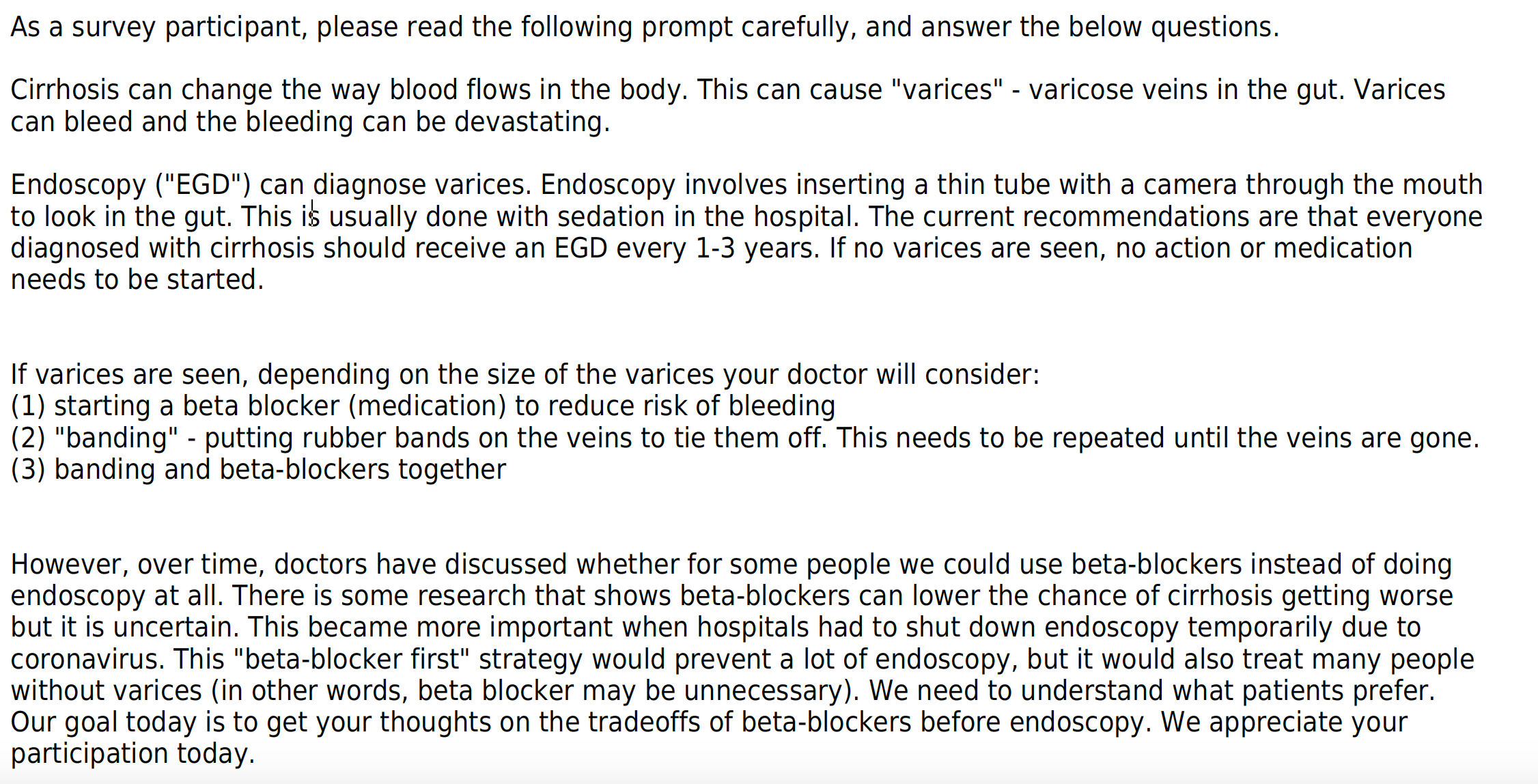


**Supplemental Figure 2.** Survey Prompt
