# Supplementary figures and images for: Pill or procedure? Patient preferences on beta-blockers as an alternative to endoscopic variceal screening during the COVID-19 pandemic
Source: Gastroenterol Rep (Oxf). 2022 May 6;10:goac015. doi: 10.1093/gastro/goac015 (PMC9073924; doi:10.1093/gastro/goac015)

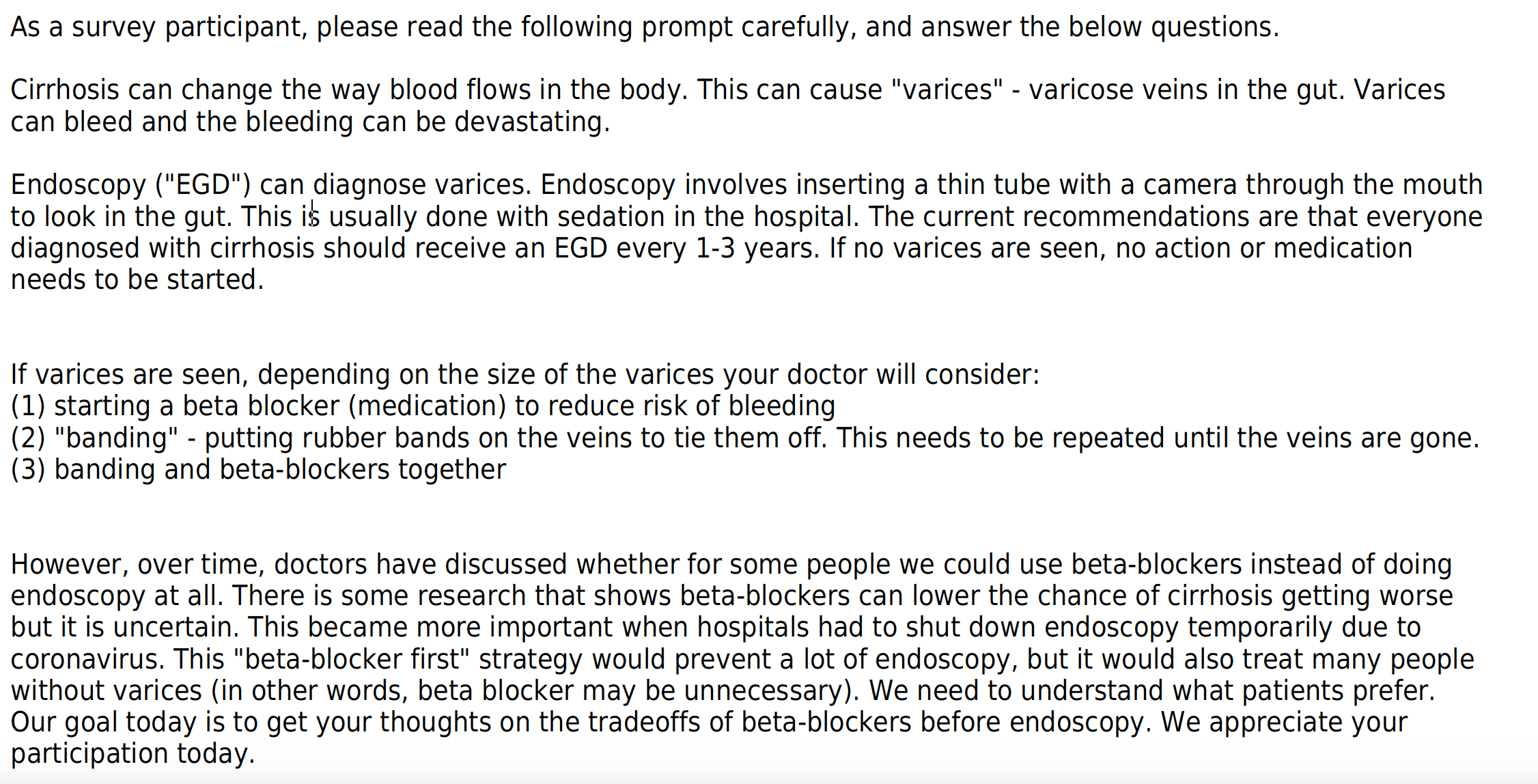

Supplement: goac015_Supplementary_Data [file goac015_supplementary_data.zip › 2022-042 Supplemental_Figure_2.tif]

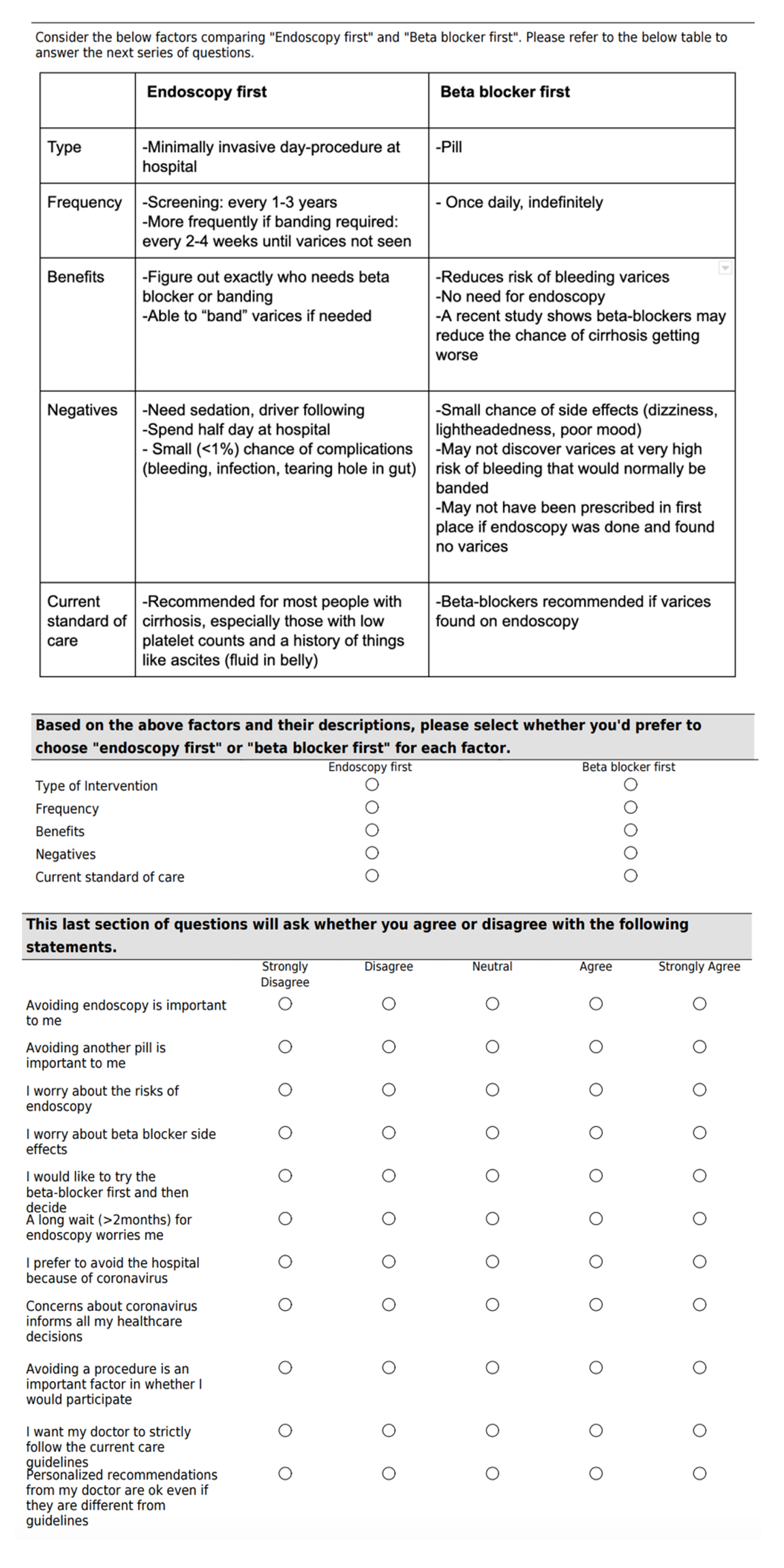

Supplement: goac015_Supplementary_Data [file goac015_supplementary_data.zip › 2022-042 Supplemental_Figure_1.tiff]
